# Starvation Ketoacidosis in Pregnancy: An Unusual Presentation and Brief Literature Review

**DOI:** 10.1210/jcemcr/luae145

**Published:** 2024-08-16

**Authors:** Lisa Abraham, Xinyuan Ning, Hilary B Whitlatch

**Affiliations:** Department of General Internal Medicine, University of Maryland Medical Center, Baltimore, MD 21201, USA; Division of Endocrinology, Diabetes, and Nutrition, University of Maryland Medical Center, Baltimore, MD 21201, USA; Division of Endocrinology, Diabetes, and Nutrition, University of Maryland Medical Center, Baltimore, MD 21201, USA

**Keywords:** starvation ketoacidosis, pregnancy, starvation ketoacidosis of pregnancy

## Abstract

Starvation ketoacidosis in pregnant patients is a rare but life-threatening condition that requires prompt diagnosis and timely treatment. A 35-year-old pregnant woman at 33 weeks’ gestation was admitted for abdominal pain with poor oral intake. She was diagnosed with perforated appendicitis and underwent emergent laparotomy. During the procedure and afterwards, she was found to have an anion gap metabolic acidosis. She was treated with a dextrose infusion with a fixed-rate insulin with correction of metabolic parameters. Women in late pregnancy are at increased risk for ketosis from increased relative insulin resistance and enhanced lipolysis. There is no consensus on optimal management of starvation ketoacidosis of pregnancy; however, carbohydrate administration is a cornerstone of treatment. We chose simultaneous administration of carbohydrates with insulin to overcome any inherent insulin resistance and to suppress lipolysis with rapid resolution of the patient's metabolic derangements.

## Introduction

Starvation ketoacidosis in pregnant women is rare but potentially life-threatening for both mother and fetus. During starvation, because of an inadequate supply of glucose to meet metabolic needs, the mother's body will adapt by increasing free fatty acid breakdown to produce ketone bodies as alternative energy sources. The degree of acidosis secondary to starvation is generally mild and not life-threatening; however, factors such as pregnancy, infection, and stress may exacerbate the severity of metabolic acidosis (1). There are only a few cases of starvation ketoacidosis in pregnancy described in the literature, with no consensus for treatment. We report an unusual case of starvation ketoacidosis in a pregnant woman secondary to perforated appendicitis that was successfully treated with dextrose and insulin infusion.

## Case Presentation

A 35-year-old woman presented at 33 weeks of gestation in her sixth pregnancy with 1 week of worsening abdominal pain that was accompanied by 3 days of vomiting with inability to tolerate oral intake. She had a history of gestational diabetes diagnosed in a previous pregnancy but not her current pregnancy. She was started on metformin at week 29 of her current pregnancy because she had failed her 1-hour glucose tolerance test; however, she refused confirmatory testing so gestational diabetes could not be confirmed. Notably, her hemoglobin A1c before this admission was noted to be 5.6% (reference range, 4.8%-5.6%).

Initial evaluation of the patient showed a blood pressure of 117/64 mm Hg, a pulse of 111 beats/min, respirations of 20 breaths/min, and a temperature of 37.1 °C. The physical examination was notable for a soft abdomen with moderate generalized tenderness to palpation and guarding in the right lower quadrant.

Her initial blood work showed a white blood cell count of 16.100 K/μL(16.1 × 10^9^/L) (reference range, 4.5-11 K/μL; 4.5-11.0 × 10^9^/L), a serum bicarbonate of 17 mEq/L (17 mmol/L) (reference range, 21-30 mEq/L; 21-30 mmol/L), and an anion gap of 15 mEq/L (15 mmol/L) (reference range, 4-16 mEq/L; 4-16 mmol/L). Her serum glucose at this time was 75 mg/dL (4.163 mmol/L) (reference range, 70-99 mg/dL; 3.9-5.6 mmol/L). Because the patient had been reporting persistent diffuse abdominal pain, an ultrasound was done, but findings were nondiagnostic and so magnetic resonance imaging of the abdomen/pelvis was obtained that showed right lower quadrant edema concerning for perforated appendicitis. The patient was taken emergently to surgery where she underwent an open appendectomy with intraoperative findings of gross purulent fluid in the peritoneum. During the operation, she was noted to have an anion gap metabolic acidosis with arterial blood gas pH of 7.06 (reference range, 7.35-7.45), pCO_2_ of 43 mm Hg (5.7 kPa) (reference range, 32-35 mm Hg; 4.4-5.9 kPa), pO_2_ of 120 mm Hg (16.0 kPa) (reference range, 83-108 mm Hg; 10.0-14.0 kPa), and bicarbonate of 12 mEq/L (12 mmol/L) (reference range, 21-30 mEq/L; 21-30 mmol/L). She was treated with 300 mEq of bicarbonate during surgery to temporize her acidosis, which improved to an arterial pH of 7.23 (reference range, 7.35-7.45) before falling again. Because of her tenuous postoperative status and persistent acidosis, she was kept intubated postoperatively.

After the operation, she remained acidotic with an arterial pH of 7.24 (reference range, 7.35-7.45). Further blood work demonstrated a serum bicarbonate of 14 mEq/L (14 mmol/L) (reference range, 21-30 mEq/L; 21-30 mmol/L), and an anion gap of 18 mEq/L (18 mmol/L) (reference range, 4-16 mEq/L; 4-16 mmol/L) with a beta-hydroxybutyrate of 58.5 mg/dL (9.59 mmol/L) (reference range, 0-3 mg/dL; 0-0.3 mmol/L). She had a serum glucose of 134 mg/dL (7.4 mmol/L) (reference range, 70-99 mg/dL; 3.9-5.6 mmol/L) and a lactate of 1.3 mEq/L (1.3 mmol/L) (reference range, 0.5-2.2 mEq/L; 0.5-2.2 mmol/L). Urinalysis demonstrated 2+ ketones (reference range, negative) and she had a “small” serum acetone value (reference range, negative).

## Diagnostic Assessment

Because of her persistent high anion gap acidosis with ketone formation without significant hyperglycemia, there was high suspicion for starvation ketoacidosis because of her gravid status. Other causes of anion gap metabolic acidosis included lactic acidosis, which was less likely given that she had a normal lactate. Concern for appendicitis and sepsis was treated with antibiotics. Electrolytes were monitored with repletion and periodic laboratory work. The patient also had normal renal, hepatic, and cardiac functions.

## Treatment

She was started on a dextrose 10% infusion at 75 mL/hour and a fixed-dose insulin infusion at 4 units per hour.

## Outcome and Follow-up

**Figure 1.**
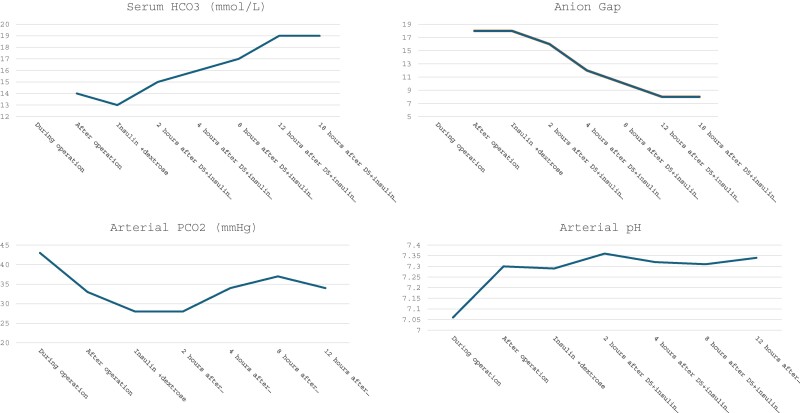
Metabolic parameters of anion gap acidosis over the course of 12 hours after starting dextrose containing fluids.

Repeat arterial blood gases and chemistry showed rapid improvement in pH and bicarbonate after administration of dextrose and insulin (Table 1 and Fig. 1). After starting treatment, the patient was extubated in 24 hours and was liberated from the dextrose and insulin infusions after 48 hours.

**Table 1.** Laboratory parameters

Laboratory values	Reference	Day 2	Day 2	Day 2	Day 2	Day 2	Day 3	Day 3	Day 3	Day 3
		During operation	After operation	Insulin + dextrose	2 h after D5 + insulin drip	4 h after D5 + insulin drip	8 h after D5 + insulin drip	12 h after D5 + insulin drip	18 h after D5 + insulin drip	23 h after D5 + insulin drip
Time		13:06	16:00	18:30	20:40	22:25	02:04	07:50	12:02	16:55
Arterial pH	7.35-7.45	7.06	7.3		7.29	7.36	7.32	7.31	7.34	
Arterial PCO_2_	32-48 mm Hg (4.4-5.9 kPa)	43 mm Hg (5.7 kPa)	33 mm Hg (4.4 kPa)		28 mm Hg (3.7 kPa)	28 mm Hg (3.7 kPa)	34 mm Hg (4.5 kPa)	37 mm Hg (4.9 kPa)	34 mm Hg (4.5 kPa)	
Arterial pO_2_	83-108 mm Hg (10.0-14.0 kPa)	120 mm Hg (16.0 kPa)	93 mm Hg (12.4 kPa)		194 mm Hg (25.9 kPa)	157 mm Hg (20.9 kPa)	160 mm Hg (21.3 kPa)	112 mm Hg (14.9 kPa)	162 mm Hg (21.6 kPa)	
Serum HCO3^−^	21-30 mEq/L (21-30 mmol/L)		14 mEq/L (14 mmol/L)		13 mEq/L (13 mmol/L)	15 mEq/L (15 mmol/L)	16 mEq/L (16 mmol/L)	17 mEq/L (17 mmol/L)	19 mEq/L (19 mmol/L)	19 mEq/L (19 mmol/L)
Anion gap	4-16 mEq/L (4-16 mmol/L)		18 mEq/L (18 mmol/L)		18 mEq/L (18 mmol/L)	16 mEq/L (16 mmol/L)	12 mEq/L (12 mmol/L)	10 mEq/L (10 mmol/L)	8 mEq/L (8 mmol/L)	8 mEq/L (8 mmol/L)
Beta-hydroxybutyrate	0-3 mg/dL (0-0.3 mmol/L)		58.5 mg/dL (3.3 mmol/L)							
Lactate	0.5-2.2 0.5-2.2 mEq/L (0.5-2.2 mmol/L)		1.2 mEq/L (1.2 mmol/L)		1.3 mEq/L (1.3 mmol/L)		0.9 mEq/L (0.9 mmol/L)		1.0 mEq/L (1.0 mmol/L)	
Glucose	70-99 mg/dL (3.9-5.6 mmol/L)		134 mg/dL (7.4 mmol/L)		117 mg/dL (6.5 mmol/L)	120 mg/dL (6.7 mmol/L)	123 mg/dL (6.8 mmol/L)	137 mg/dL (7.6 mmol/L)	147 mg/dL (8.2 mmol/L)	125 mg/dL (6.9 mmol/L)
Urine ketones	Negative		+2		+2		+2			
IV fluid		Bicarb IV 300 mEq	Dextrose 5%, ½ NS 125 mL/h	Insulin regular 4 U/h, Dextrose 5%, ½ NS 125 mL/h	Insulin regular 4 U/h, Dextrose 5%, ½ NS 125 mL/h	Insulin regular 4 U/h, Dextrose 5%, ½ NS 150 mL/h	Insulin regular 4 U/h, Dextrose 5%, ½ NS 150 mL/h	Insulin regular 4 U/h, Dextrose 5%, ½ NS 200 mL/h	Insulin regular 5 U/h, Dextrose 5%, ½ NS 200 mL/h	Insulin regular 5 U/h, Dextrose 5%, ½ NS 150 mL/h

Values in parentheses are International System of Units (SI).

Abbreviations: Bicarb IV, bicarbonate drip; dextrose 5%; ½ NS, 5% dextrose solution and half normal saline.

She was stabilized and then eventually underwent a cesarean section 10 days later because she developed pelvic abscesses from the ruptured appendicitis requiring surgical intervention. She delivered a healthy baby girl. Her requirement for a cesarean section was not related to her starvation ketoacidosis, which had been resolved for more than a week before the procedure. Both mother and baby were discharged in good condition.

## Discussion

Starvation ketoacidosis is a condition in which there is accumulation of ketone bodies that occurs because of inadequate glucose supply to meet metabolic demand. During starvation, ketone bodies are synthesized in the liver as cells shift from glucose to lipid metabolism producing acetoacetate, beta-hydroxybutyrate, and acetones. Acetoacetate and beta-hydroxybutyrate are then used as alternative energy sources; however, their accumulation can result in metabolic acidosis (2). Insulin plays an important role in inhibiting lipolysis and ketogenesis. Insulin deficiency, either from absolute deficiency, as in people with type 1 diabetes mellitus, or relative deficiency, as in women in late stages of pregnancy, are more prone to develop ketoacidosis.

A relatively short period of starvation in pregnancy can precipitate ketosis through a phenomenon known as “accelerated starvation” resulting from the metabolic changes that occur during pregnancy to ensure adequate nutrition for the growing fetus. Elevated placental hormones, such as human placental lactogen, prolactin, and cortisol, which increase insulin resistance, allow serum glucose to be preferentially shunted to the growing fetus by lowering the threshold at which the mother uses lipid metabolism for energy production (1). Additionally, changes in lung volume, altered compliance, and central respiratory stimulation by progesterone lead to hyperventilation and respiratory alkalosis (3, 4). Chronic respiratory alkalosis is compensated through renal bicarbonate excretion, which results in lower plasma bicarbonate concentration and a reduced buffering capacity, which compounds the increased risk for acidosis in later trimesters.

The exact prevalence for starvation ketoacidosis in pregnancy is not known and because of the relative rarity of the diagnosis, most of what is known on diagnosis and treatment is gathered from a compilation of case reports. Most cases occur in the third trimester of pregnancy and are usually preceded by poor oral intake along with increased nausea and vomiting over several days. Cerere et al does describe a patient who developed starvation ketoacidosis with less than 24 hours of decreased oral intake with vomiting (Table 2) (5).

**Table 2.** Summary of previous cases

Reference	Presentation	Initial pH	Comorbidity	Treatment	Outcome
Darbhamulla et al (6)	30-y-old at 33 wk presenting with coffee ground emesis × 24 h	7.17	Gestational diabetes, not on insulin	D10 infusion Sliding scale insulin	Elective cesarean section at 39 wk
Land et al (7)	37-y-old at 32 wk presenting with nausea/vomiting/malaise for less than 24 h	7.02	Preterm labor	D5 infusion	Intrauterine demise
Mahoney (8)	37-y-old at 30 wk presenting with preterm labor	7.22	Preterm labor, gestational diabetes not on insulin	D10 infusion with fixed rate insulin	Emergency cesarean at 30 wk
Keay and Fox (9)	22-y-old at 35 wk presenting with vomiting × 5 days	7.23	None	Sliding scale insulin with D10 infusion	Emergency cesarean at 35 wk
Burbos et al (10)	39-y-old woman at 33 wk presents with vomiting × 24 h	7.19	None	D10 infusion	Spontaneous labor postadmission
Patel et al (3)	29-y-old at 32 wk presenting with vomiting × 1 week	7.215	None	D5 infusion, delivery of baby	Emergency cesarean at 33 wk
Frise et al (1)	4 cases, age range 22-40 at range of 29-35 wk all presenting with vomiting × 24+	7.27-7.29	1 patient with gestational diabetes	None, delivery of baby	Emergency cesarean in all 4 cases
Scholte and Boer (11)	26-year-old at 35 wk, 4 days presents with abdominal pain and dyspnea	7.3	Twin pregnancy	D5 infusion, delivery of babies	Emergency cesarean
Karpate et al (12)	25-year-old at 37 wk, 5 days’ gestation admitted with vomiting and oral intake intolerance	—	None	D10 infusion with fixed-rate insulin	Spontaneous labor with forceps delivery
Sinha et al (4)	41-y-old woman at 32 wk gestation presenting with vomiting × 4 days	7.158	None	D5 infusion in LR	Spontaneous labor postadmission
Cecere et al (5)	31-y-old woman at 32 wk, 3 days’ with nausea and vomiting × 24 h	7.06	None	D10 infusion	Emergency cesarean
Chausse et al (13)	24-y-old at 36 wk, 2 days’ gestation presented with malaise and lower abdominal pain × 2 days and 1 day of vomiting	7.12	None	D10 infusion	Spontaneous delivery postadmission
Pikovsky et al (14)	34-year-old at 36 wk presented with shortness of breath × 3 days and anorexia × 2 wk	7.25	SARS-COVID pneumonia	D10 infusion, IV insulin infusion	Expedited cesarean section

In certain cases, an underlying infection may be present. Pivovsky et al describe a case of a patient presenting with a 2-week history of anorexia and COVID pneumonia (14). The presentation was consistent with starvation ketoacidosis resulting from poor oral intake and rapid resolution of metabolic acidosis with dextrose infusion (14).

The prompt recognition of starvation ketoacidosis of pregnancy is important because it can be easily reversed. Frise et al presented 4 cases, most of which presented with nausea and vomiting and who were started on lactated Ringer’s solution (1). Ultimately, metabolic parameters failed to improve and the patients were treated with betamethasone for preterm delivery and underwent emergency cesarean sections (1). After delivery, their symptoms resolved (1). However, emergency delivery puts the mother at risk and the fetus is often preterm (1). The lack of recognition of ketosis was part of the reason for not starting dextrose-containing fluids (1).

Scholte describes a patient admitted for hyperventilation who developed worsening respiratory distress (11). Because of concern for decompensation, a cesarean section was performed (11). After delivery, it was clear that the patient had decreased oral intake and vomiting before presentation. A 5% dextrose infusion was started, and the acidosis resolved.

Mahoney reports a case of preterm labor. During admission a 3-hour glucose tolerance test was positive (8). The patient had stable blood glucose, but later developed a severe metabolic acidosis with ketonuria (8). Because the cause of acidosis was unclear, emergency cesarean section was performed (8). After delivery, serum ketones returned positive, and 10% dextrose solution (D10) was started, along with fixed-rate insulin later with resolution of metabolic parameters (8). The delay in diagnosis of starvation ketoacidosis was partly the result of low pH not usually seen with starvation ketosis (8). This is true in nonpregnant individuals. The physiologic changes of pregnancy cause an exaggerated response, leading to more severe acidosis in starvation in pregnancy, which may also have been exacerbated by relative insulin resistance from her gestational diabetes (8).

Land et al demonstrates the severity of nondiabetic ketoacidosis, which caused intrauterine fetal demise. The patient received salbutamol and developed nausea and vomiting (7). The patient had severe acidosis and low blood glucose, and the patient was started on a 5% dextrose solution (D5) infusion. After delivery, arterial pH normalized (7).

Most treatment described in case reports involves dextrose infusion, insulin, and emergency cesarean section. In Table 2, several case reports involve the use of D5. Although D5 is commonly used in clinical practice, it is hypothesized that because of the low glucose concentration, the dextrose is rapidly metabolized and the fluid does not stay in the intravascular compartment (1). The low glucose content in D5 does not sufficiently inhibit lipolysis enough to reverse acidosis.

In 2 of 3 cases in which D5 was used, patients had to undergo emergency cesarean sections (3, 13). Land et al describe a patient who was started on a D5 infusion but ultimately resulted in intrauterine demise (7).

Although 50% dextrose is used for hypoglycemia, if given peripherally, it can be an irritant. Frise et al recommend a D10 because it provides sufficient dextrose to inhibit lipolysis and enough volume for volume resuscitation (1). If glucose increases with D10 and ketones remain elevated, the addition of insulin is recommended (1, 12). D10 allows more provision of carbohydrates than D5, which allows for endogenous insulin production and the inhibition of further ketone production allowing the ketosis to resolve (8, 12).

The use of insulin is also recommended, Karpate et al recommend the use of fixed-dose insulin, for ketogenesis suppression (12).

Pikovsky et al recommend confirming the presence of ketones with acidosis and normoglycemia to diagnose starvation ketoacidosis and recommend using fixed-rate insulin with dextrose as treatment (14).

Bicarbonate can also be useful. The acidosis can be exacerbated by decreased buffering capacity resulting from chronic respiratory alkalosis and renal compensation causing bicarbonate excretion (3). Patel also recommended that a dextrose infusion of less than 20 g/hour of IV dextrose should be used (3).

In our case, we started a fixed rate insulin infusion with D10 infusion (Table 1). Note that the patient was intermittently on D5 infusion at double rate for fluid resuscitation (Table 1).

On literature review, most patients were treated with D10 with or without fixed-rate insulin (Table 2). D5 appears to be unfavorable, resulting in emergency cesarean sections and was associated with the only intrauterine demise. Starting D10 is recommended and if there is concern for marginal insulin reserve or if metabolic parameters do not improve, starting fixed-rate insulin is recommended to further suppress ketosis.

Starvation ketoacidosis is underrecognized. Carbohydrate administration is the cornerstone of treatment. Insulin administration with close glucose monitoring can be considered if there is a concern for marginal insulin reserve or treatment failure. The prognosis of starvation ketoacidosis remains good; however, prompt diagnosis and early intervention is critical in preventing further deterioration and fetal morbidity and mortality.

## Learning Points

- Starvation ketoacidosis is underrecognized and prompt recognition and management are essential in avoiding maternal and fetal mortality
- Carbohydrate administration, in the form of D10, is the cornerstone for treatment for starvation ketoacidosis
- Starting fixed-rate insulin is also recommended to further suppress ketosis if there is concern for marginal insulin reserve or if metabolic parameters do not improve.

## Contributors

All authors made individual contributions to the authorship. L.A. and X.N. conceived the manuscript. L.A. wrote the manuscript with contributions from X.N. and H.W.

## Data Availability

Data sharing is not applicable to this article as no datasets were generated or analyzed during the current study.

## Abbreviations

D5: 5% dextrose solution
D10: 10% dextrose solution
